# Mobile applications (apps) for tobacco cessation: Behaviour change potential and heuristic analysis using the Mobile Application Rating Scale (MARS)

**DOI:** 10.12688/f1000research.142038.2

**Published:** 2024-11-22

**Authors:** Bhargav Bhat, Prajna Pramod Nayak, Ramprasad Vasthare, Deepak Kumar Singhal

**Affiliations:** 1 Department of Public Health Dentistry, Manipal College of Dental Sciences, Manipal Academy of Higher Education, Manipal, Karnataka, 576104, India

**Keywords:** smoking cessation, mobile health, mHealth, tobacco use cessation, mobile app, oral health

## Abstract

**Background:**

Given the high prevalence of tobacco use, India presents a significant challenge in tobacco control. Yet, the support received for tobacco cessation is suboptimal. Hence, the aim of this narrative review was to identify and heuristically evaluate ‘high-quality’ and ‘engaging’ tobacco cessation mobile apps using the Mobile Application Rating Scale (MARS). Also, to categorize and analyse their features with respect to engagement, functionality, aesthetics, and information quality.

**Methods:**

A systematic search of tobacco cessation apps was done within app stores of prominent smartphone platforms developed by Apple and Android. The following search terms: ‘quit smoking,’ ‘smoking cessation, ‘stop smoking,’ ‘smoking therapy,’ ‘quit tobacco,’ ‘cigarette cessation,’ ‘cold turkey,’ and ‘quit cigarette.’ Pearson’s correlations were used to analyse correlations between app scores (Total score app-quality/mean score) and downloads/ratings and number of downloads with the overall MARS score. A Chi-square test was performed to analyse any association between app focus and app release dates.

**Results:**

Total MARS scores ranged from 3.1 to 4.9. Quitsure app (4.9), Craving to Quit! app (4.8) and Stop Tobacco Mobile Trainer: Quit Smoking App (4.74) were ranked the highest according to the MARS overall mean score. Older apps focussed more on mere goal setting or substance use, as compared to behaviour change; whereas, recently developed apps are now focussing more on behaviour change.

**Conclusions:**

The content and functionality of behaviour change-focused apps were of higher quality than those of other app categories. These recently developed mHealth apps can effectively supersede the traditional smoking cessation methods.

## Introduction

Tobacco use is the world’s most significant threat that is responsible for numerous preventable morbidities, most of which have the potential to kill consumers prematurely. Six of the top eight causes of mortality have smoking as a risk factor. The 20th century has seen an estimated 100 million fatalities worldwide as a result of the tobacco epidemic. ^1^ India is currently experiencing a second-stage tobacco pandemic. Each year, 800,000 to 900,000 people in India die from diseases linked to tobacco use. It is crucial to act swiftly to stop this preventable disease. ^2^

Given the high prevalence of tobacco use, India represents a significant challenge in tobacco control. According to the latest Global Adult Tobacco Survey (GATS) India (2016–17), 28.6% of smokers in India use tobacco, with male smokers having a greater prevalence (42.4%) than female smokers (14.2%). According to this nationally representative survey, 52% of tobacco users are ready to give up the habit. ^3^^,^^4^ Tobacco cessation programs run at hospitals and rehabilitation centres provide face-to-face and telephone-based behavioural interventions (e.g., brief motivational advice, group or individual counselling, telephone counselling and quit lines) and pharmacotherapy (e.g., nicotine replacement therapies, bupropion) to support smoking cessation. Yet, tobacco cessation support is suboptimal owing to the lack of access to smoking cessation resources, lack of clinician knowledge or skills, and lack of clinician time. Most people making independent attempts fail to maintain their quit status for even one month. ^2^ Hence, use of mHealth intervention for smoking cessation represents one of the best methods to curb the global public health threat of the tobacco epidemic. ^5^

In the recent times, mobile phones, the Internet and mobile communication have become an inevitable part of our lives. According to estimates, there are 751.5 million internet users in India by the beginning of 2024, with internet penetration of 52.4%. In early 2024, India had 1.12 billion active cellular mobile connections, which accounted for 78% of the entire population, providing a sizable number of users to help conduct mobile interventions to distribute information. ^6^ An “app” is the most frequent term for a mobile application that is created to run on portable wireless mobile phones like smartphones. ^7^^,^^8^ With over three billion downloads of mHealth apps worldwide in 2015, an effective, high-quality smoking cessation app can be an easy-to-use resource that can provide personalized smoking cessation support to thousands of people, including remote smokers, at the right time and location. Past reviews show that mobile apps have tremendous behaviour change potential. ^7^^–^^9^

The utilization of mobile applications in healthcare can offer a range of benefits, including ease of access to information and the potential for enhanced patient engagement and adherence to treatment. However, there are some drawbacks to the use of mobile applications, particularly as the information on apps may not be regulated. As a result, some of the apps that patients access may contain substantial inaccuracies. ^8^

The use of smoking cessation interventions utilizing smartphone technology is on the rise globally. However, the effects on cessation rate and prevention of relapses are not often evaluated. ^10^ Moreover, few studies are reporting the effectiveness of smartphone apps for tobacco cessation. What’s more, rankings within the leading app stores are highly volatile and primarily based on the usability of apps. Hence, the behaviour change potential of apps cannot be solely judged based on app ‘ratings’ or ‘popularity’ in app stores.

**Figure 1.**
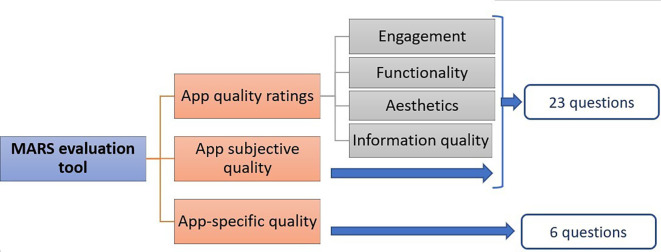
Mobile Application Rating Scale (MARS) description.

Recently many evaluation criteria/scales have been developed to evaluate app efficacy. Evaluation based on addressing the 5 As (Ask, Advise, Assess, Assist and Arrange) of tobacco cessation, App Behaviour Change Scale (ABACUS), adherence to clinical practice guidelines for tobacco control, Mobile Application Rating Scale (MARS) have been commonly used in recent studies. ^11^^–^^15^ The latter evaluation method, MARS was developed by Stoyanov SR, is widely used and validated by many studies ( Figure 1). ^15^^–^^18^ This 23-item rating measure assesses the quality of mHealth apps on a 5-point scale (1=inadequate, 2=poor, 3=acceptable, 4=good, and 5=excellent). Hence, we aimed to identify and heuristically evaluate ‘high-quality’ and ‘engaging’ tobacco cessation mobile apps using MARS. The objectives were to identify tobacco cessation apps on Apple and Android app stores and to categorize and analyze their features as regards to engagement, functionality, aesthetics, and information quality. Thereby recommending directions for the improvement of these apps.

## Methods

### Ethics statement

The study was registered with Kasturba Hospital ethics committee, approval number [IEC2 – 19 (2022), dated 21/06/2022.

### Search strategy

A systematic search of tobacco cessation apps was done within the Apple and Android app stores. The following search terms were used: ‘smoking cessation, ‘quit smoking,’ ‘stop smoking,’ ‘smoking therapy,’ ‘quit tobacco,’ ‘cigarette cessation,’ ‘cold turkey,’ and ‘quit cigarette.’ All the apps rated 4* and above, with downloads of more than 10,000 were included in the study. Apps that did not target tobacco cessation and those not in English were excluded from the study. We also eliminated the duplicate apps, including earlier versions. To begin with, we identified all apps based on their focus area:

• Substance use: information about the ill-effects of tobacco and thereby motivating users to quit.
• Goal setting: monitoring and tracking the process of quitting.
• Behaviour change: using therapeutic techniques such as cognitive behaviour therapy (CBT), acceptance commitment therapy (ACT) and mindfulness.

Subsequently, extracted data from each app were assessed using the MARS rating scale. The MARS evaluation tool is divided into three broad sections: App overall quality, App subjective quality, and App specific quality. It consists of 23 questions designed to help analyse the mobile applications' engagement, functionality, aesthetics, information, and subjective quality. In addition, there are six additional app-specific questions that can be customized to represent the goal health behaviour/function of the application/study. The average scores for each of these four sub-schemes were calculated, and the average scores of these sub-quotas used to evaluate the overall quality score of the application ranged from 1 to 5. Therefore, the total score of these sub-categories would be 1 to 5 (1=inadequate, 2=poor, 3=acceptable, 4=good, and 5=excellent) and the average score would be calculated by dividing the total score by 4, i.e., four domains. The subjective quality section of the App and the App-specific section of the App were calculated similarly. These, however, are considered as separate from the app quality score ( Figure 1).

Data regarding the App’s features were entered in a Microsoft Excel sheet v 2019 and subjected to statistical analysis to conclude. The two reviewers were trained in the use of MARS using a 37-minute video on YouTube (MARS training video: https://www.youtube.com/watch?v=25vBwJQIOcE). The study team also established specific instructions and guidelines for assessing smoking cessation apps so as to ensure consistency between the raters. Subsequently, they conducted an independent assessment of a standard application and discussed their scores. The quality scores were then calculated as an average of the ratings from the two reviewers.

### Statistical analysis

Data were analysed using descriptive and analytical statistics. Quantitative variables were expressed as means and standard deviations. Kendall’s coefficient of concordance was used to calculate the interrater agreement between two raters. Pearson’s correlations were used to analyse correlations between app scores (Total score app-quality/mean score) and downloads/ratings and number of downloads with the overall MARS score. A Chi-square test was performed to analyse any association between app focus and app release dates with a p-value of <0.05 considered as significant.

## Results

**Figure 2.**
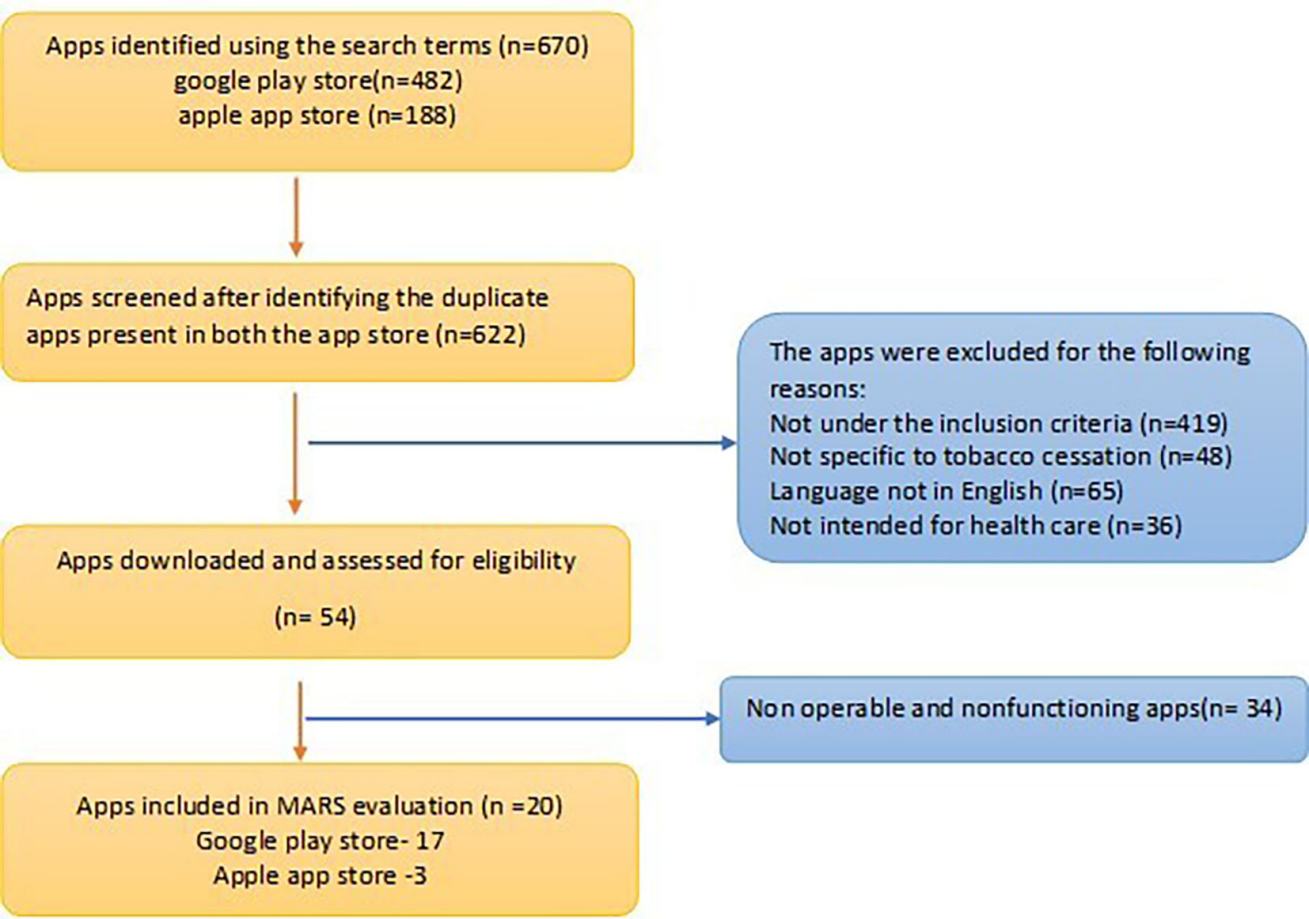
Flowchart for identification of Apps through systematic screening of the available tobacco cessation apps.

A total of 670 apps were retrieved from within the Apple and Android App stores (Android 482; iOS 188). After screening the description available on the web page, 48 apps were excluded as they were duplicate apps present in both the Play stores. ^19^ Of the 622 apps filtered, 568 apps didn’t meet the inclusion criteria and were excluded from the study. After all the exclusions, 20 apps (Android,17; iOS, 3) were subjected to analysis. A flowchart illustrating the selection and exclusion of apps at various stages of the study is shown in the Figure 2. Interrater agreement between two raters showed high reliability (0.804).

All apps were free in their basic version, but 14 required payments to view their upgraded versions. As per their primary focus area, two apps targeted only substance use, while 14 apps focused on goal setting and four apps were aimed at behaviour change. Five apps had an app-based community that allowed virtual help. All apps were available for general age groups ( Table 1). Fifteen apps had second or higher versions and the last update of most apps was after June 2023. The mean app rating by consumers in the Google Play store was more than 4 (according to the inclusion criteria) ( Table 2).

**Table 1.** Basic attributes of mHealth apps for tobacco cessation.

Characteristics of the app		Apps n (%)
Platform	Android	17
	iOS	3
Cost	Free upgraded versions	6
	Paid upgraded versions	14
Year of release	2010-2014	10
	2015-2018	5
	2019-2022	5
Number of downloads	Up to ten thousand	4
	Up to one million	12
	More than one million	4
Primary focus	Substance use	2
	Goal setting	14
	Behaviour change	4

**Table 2.** General attributes of apps included in the study.

No.	App name	Rating	Last update	Cost	Legitimate source
1	Smoke Free- Stop Smoking Now	4.6	06-11-2024	Paid	Source identified - Individual
2	Quitsure	4.7	21-10-2024	Paid	Source identified - Individual
3	Quit Now: Quit Smoking for Good	4.6	05-11-2024	Paid	Source identified - University
4	Stop Tobacco Mobile Trainer. Quit Smoking App	4.5	22-06-2022	Paid	Source identified - University
5	Easy Quit Stop Smoking	4.7	15-08-2024	Paid	Source not identified
6	Quitzilla: Bad habit tracker	4.6	14-10-2024	Paid	Source not identified
7	Smoking Log	4.4	25-09-2023	Free	Source not identified
8	Quit Now: My Quitbuddy	4.7	03-10-2024	Free	Source identified – Government funded
9	Craving To Quit!	4.4	11-09-2024	Paid	Yes; source identified - University
10	Quit It- Stop Smoking Today	4.6	17-06-2019	Paid	Source not identified
11	Quit Tracker: Stop Smoking	4.7	02-09-2024	Paid	Source not identified
12	Smokefree: Quit smoking slowly	4.5	04-11-2023	Paid	Source not identified
13	Kwit- Quit Smoking for Good	4	07-10-2024	Paid	Source not identified
14	Quitstart	4.3	16-05-2023	Free	Source identified – Government funded
15	Stop smoking - Stay sober	4.2	02-08-2024	Paid	Source not identified
16	No Smoking	4.4	20-11-2023	Paid	Source not identified
17	Qwit	4.1	03-10-2023	Free	Source not identified
18	Quit Smoking cigarette - Smoxy	4.6	05-11-2024	Paid	Source not identified
19	Quit Smoking- Get Healthy	4.7	25-08-2023	Free	Source not identified
20	Quit Smoking	4.5	10 months ago	Free	Source not identified

Table 3 gives the mean app quality ratings scored under engagement, functionality, aesthetics and information domains. The Craving To Quit! and QuitSure apps recorded a score of 4.75 and above in all four domains. Whereas, the SmokeFree: Quit smoking slowly and Qwit apps had scores of 3.7 or below in all the domains. The average score of the above four domains, that is the App quality mean score, was highest for the QuitSure app (4.88) and Craving To Quit! app (4.83).

**Table 3.** Mean app quality ratings scored under engagement, functionality, aesthetics and information domains.

No.	App name	Engagement mean score	Functionality mean score	Esthetics mean score	Information mean score
1	Smoke Free- Stop Smoking Now	4.4	4.75	4.67	4.4
2	Quitsure	5	4.75	5	4.8
3	Quit Now: Quit Smoking for Good	4.2	4.75	5	4.2
4	Stop Tobacco Mobile Trainer. Quit Smoking App	4.6	4.75	5	4.6
5	Easy Quit Stop Smoking	3.6	3.75	3.67	3.6
6	Smokefree-stop smoking now	3.6	4	4	4
7	Smoking Log	3.4	4	3.67	3.4
8	Quit Now: My Quitbuddy	4.6	4.5	4.67	4.6
9	Craving To Quit!	4.8	4.75	5	4.8
10	Quit It- Stop Smoking Today	3.4	3.75	3.33	3.6
11	Quit Tracker: Stop Smoking	4.2	4.25	4.67	4
12	Smokefree: Quit smoking slowly	3.5	3.5	3.33	3
13	Kwit- Quit Smoking for Good	4.8	4.5	4.67	4.8
14	Quitstart	4.2	3.75	4	3.8
15	Stop smoking - Stay sober	4	4	4	3.6
16	No Smoking	3.8	3.75	3.33	3.6
17	Qwit	3.4	3.5	3.67	3.4
18	Quit Smoking- Stop Tobacco Mobile Trainer	4.4	4.5	4.67	4.2
19	Quit Smoking- Get Healthy	4	4	4	3.6
20	Quit Smoking	3.8	4	4	3.6

The MARS overall mean score was then calculated by calculating an average of App quality mean score, App subjective mean score and App specific mean score. We observed that the QuitSure app (4.9), Craving To Quit! app (4.8) and Stop Tobacco Mobile Trainer: Quit Smoking App (4.74) were ranked the highest according to the MARS overall mean score. Whereas, Smokefree: Quit smoking slowly (3.1) and Qwit (3.2) scored the least ( Table 4).

**Table 4.** The scores of each Mobile Application Rating Scale (MARS) domain and overall MARS score of the studied apps.

No.	Android app name	App quality mean score	App subjective mean score	App specific mean score	MARS overall mean score
1	Smoke Free- Stop Smoking Now	4.55	4.25	4.33	**4.37**
2	Quitsure	4.88	5	4.83	**4.9**
3	Quit Now: Quit Smoking for Good	4.53	4	4.16	**4.23**
4	Stop Tobacco Mobile Trainer. Quit Smoking App	4.73	5	4.5	**4.74**
5	Easy Quit Stop Smoking	3.65	3	3.5	**3.38**
6	Smokefree-stop smoking now	3.9	3	3.66	**3.52**
7	Smoking Log	3.61	3	3.66	**3.42**
8	Quit Now: My Quitbuddy	4.59	4.25	4.66	**4.5**
9	Craving To Quit!	4.83	4.75	4.83	**4.8**
10	Quit It- Stop Smoking Today	3.52	3	3.33	**3.28**
11	Quit Tracker: Stop Smoking	4.27	4.25	4.16	**4.23**
12	Smokefree: Quit smoking slowly	3.35	3	3	**3.11**
13	Kwit- Quit Smoking for Good	4.69	4.75	4.66	**4.7**
14	Quitstart	3.93	3.5	3.83	**3.75**
15	Stop smoking - Stay sober	3.9	3.5	3.66	**3.68**
16	No Smoking	3.62	3.5	3.33	**3.48**
17	Qwit	3.49	3	3.16	**3.21**
18	Quit Smoking- Stop Tobacco Mobile Trainer	4.44	4.75	4.5	**4.56**
19	Quit Smoking- Get Healthy	3.9	3.75	3.66	**3.77**
20	Quit Smoking	3.85	3.75	3.83	**3.81**

We examined if there was any correlation between average review scores and overall MARS scores. There was a moderate, positive correlation seen, which was statistically significant (r = 0.395, n = 20, p = 0.038). Also, a moderate, positive correlation between the number of downloads and the overall MARS score was seen (r = 0.480, n = 20, p = 0.013) ( Table 5).

**Table 5.** Correlation between user ratings and MARS overall score.

		User ratings	MARS ratings
User ratings	Pearson Correlation	1	.395 ^*^
	Sig. (2-tailed)		.038
MARS rating	Pearson Correlation	.395 ^*^	1
	Sig. (2-tailed)	.038	

* Correlation is significant at the 0.05 level (2-tailed).

A Chi-square test was performed to analyse any association between app focus and app release dates. Older apps focused more on mere goal setting or substance use, as compared to behaviour change. Apps that have been recently developed are now focusing more on behaviour change and these results were statistically significant (P value = 0.000) ( Table 6).

**Table 6.** Association between app focus and app release dates.

	Release year		
Focus	2011-2014 (%)	2015-2018 (%)	2019-2022(%)
**Behavior change**	0 (0%)	1 (25%)	3 (75%)
**Substance use**	1 (50%)	1 (50%)	0 (0%)
**Goal setting**	9 (64.3%)	3 (21.4%)	2 (14.3%)

## Discussion

This study reviewed mobile applications for quitting smoking and used the MARS scale to rate the apps' quality. It is one of the most popular scales for evaluating the value and content of mHealth apps. It is a multidimensional tool for assessing the quality using semantic analysis and synthesis of existing literature. ^15^ The initial validation study demonstrated strong objectivity and reliability for the overall scale and subscales. ^16^^,^^17^ The MARS' applicability for quality assessment was proved by a validation study with metric evaluation. ^17^ As a result, MARS might be utilized to make the quality of apps transparent to patients and healthcare stakeholders.

Our study found more than 622 smoking cessation apps in both platforms. It is concerning that the app stores for smoking cessation applications is saturated with many apps, perhaps making it difficult for users who aren't the most selective to find higher-quality apps. There were many apps with ratings above 4 stars, we included those with a rating of 4 stars or higher as well as more than 10,000 downloads in our inclusion criteria, since apps with high ratings but very few downloads could not be relied upon. This study reviewed 20 of these apps that aimed at helping people quit smoking in order to evaluate their quality and distinguishing characteristics.

In this study, we classified apps based on their primary goals: goal setting, substance use, and behaviour change. Most of apps with the primary focus as goal setting functioned as trackers for cravings, calendars, number of cigarettes not smoked and the amount of money saved. These results are similar to the study conducted by Vilardaga *et al*., ^9^ and Hoeppner *et al*. ^11^

The results of our study are consistent with the results of earlier reviews. Education and behavioural methods are the two areas that most frequently appear in mHealth apps. ^5^^,^^9^^,^^18^ By changing their behaviour, people can better their prognosis, lessen pain or suffering, and take control of their health. According to the findings of our study, Quitsure app, Craving to quit, Kwit-quit smoking for good and Quit Now: Quit Smoking for Good apps, which focused on behaviour modification had higher user ratings and overall MARS scores with many features. In a study conducted by Thornton L et al (2017), My Quit buddy and Quitstart apps showed the highest MARS scores among 6 apps rated. ^13^ The scores of these apps were consistent with our findings. Another study conducted by Seo S et al (2023) showed similar results, where, Quitstart app showed moderately high score and My Quit buddy app obtained highest score. ^14^

Goal-setting and substance use are two additional crucial components of tobacco cessation applications. These apps function by asking users to enter their cigarette consumption before creating their own objective goals for quitting. The app also offers resources and data to help users track their progress towards their goals and maintain their motivation to stop using tobacco. However, it has been noted that the number of goal-setting apps has dropped over time.

Our study thus points to a potential market for developing behaviour change targeting apps that offer knowledge and expertise for controlling the quitting process. Therapeutic approaches like cognitive behavioral therapy (CBT), acceptance and commitment therapy (ACT), and mindfulness practices need to be incorporated while developing such apps. Like in previous studies, apps that utilize behaviour change and cognitive techniques, which are evidence based, such as the 5 A’s, acceptance and commitment therapy and hypnosis were having the highest MARS score among all the apps. ^5^^,^^13^^,^^14^ CBT is an effective approach, as it helps individuals identify and change the thoughts, behaviors, and triggers that contribute to their smoking habit. CBT helps individuals recognize the specific situations, feelings, or thoughts that trigger the urge to smoke and includes teaching coping skills to replace smoking with healthier behaviors. ACT is based on the idea that struggling against negative emotions, thoughts or experiences often leads to greater distress, avoidance and unhelpful behaviors. Instead of aiming to eliminate painful feelings, ACT encourages individuals to accept them while still acting toward meaningful life goals. ^12^^,^^13^

The aesthetic and information subscales had slightly lower mean scores than the engagement and functionality subscales, which shows that even though the apps had good graphics, a pleasing visual appearance, and accurate descriptions and information, it is still crucial that the target audience is well-engaged and is persuaded to use the apps. The results of previous studies using MARS for quality assessment of mobile apps for the management of tinnitus ^20^ and asthma management ^21^ showed the engagement and aesthetic scores were lower, which indicates that these factors are less important in the design of health management apps. These noticeable features could be improved and developed in the next version of the apps.

Generally, while creating an app, developers must take into account both intriguing and crucial features as well as high-quality, fact-based material. The typical mean scores for engagement and functionality point to prospective areas that could be improved. Further research like randomised controlled trials need to be done to determine whether these apps result in behaviour change, and not just improvement in knowledge.

This study contains some limitations. First, popularity is the basis for app store rankings. As a result, the top apps that each search displayed did not always represent the best apps. We did not have access to numerous international apps created exclusively for use within each nation as phone numbers were required for registration and app usage. Inclusion of apps with over 10,000 downloads might have excluded newer but less popular apps which are equally virtuous. Applications go through upgrades and modifications frequently. Several apps assessed may have been upgraded to newer versions since the MARS evaluation was conducted. The most recent version and these updates could change the outcome of this research. Also, given that the apps were only used once and the quality ratings were based on brief usage, it's probable that some features were overlooked by the reviewers while evaluating the apps. The strength of our study is that the raters paid for the upgraded version of a number of apps to access the full version so as to not miss any distinguishable features of those apps.

## Conclusions

The content and functionality of behaviour change-focused applications were of higher quality than those of other app categories. These recently developed mHealth apps can effectively supersede the traditional smoking cessation methods. This study can be a reference for those who use and create smoking cessation apps. Users can select the best smoking cessation app for their needs by using the classification of the application.

## Data Availability

Open Science Framework: Underlying data for ‘Mobile applications (apps) for tobacco cessation: Behaviour change potential and heuristic analysis using the Mobile Application Rating Scale (MARS)’, https://www.doi.org/10.17605/OSF.IO/JNBPF. ^19^ Data are available under the terms of the Creative Commons Attribution 4.0 International license (CC-BY 4.0)
