# Supplementary material for: Mobile applications (apps) for tobacco cessation: Behaviour change potential and heuristic analysis using the Mobile Application Rating Scale (MARS)
Source: F1000Res. 2024 Nov 22;12:1413. Originally published 2023 Oct 26. [Version 2] doi: 10.12688/f1000research.142038.2 (PMC12107232; doi:10.12688/f1000research.142038.2)
Supplement: Peer review history

# Reviewer report: Reviewer response for version 2

**DOI:** 10.5256/f1000research.174893.r380805

**Published:** 2025-05-26

**Authors:** Jinsong Chen

**Affiliations:** 1 The University of Auckland, Auckland, Auckland, New Zealand

This study presents a heuristic evaluation of tobacco cessation mobile applications using the Mobile Application Rating Scale (MARS). A total of 670 apps were identified, from which 20 high-rated apps (≥4 stars and ≥10,000 downloads) were selected and analyzed across four quality domains: engagement, functionality, aesthetics, and information. The top-performing apps— **QuitSure**, **Craving To Quit!**, and **Stop Tobacco Mobile Trainer**—were found to excel in behaviour change potential. The authors categorized apps by focus (substance use, goal setting, and behaviour change), highlighted the increasing trend of behaviour-change-oriented apps, and used appropriate statistical tools to correlate app characteristics with MARS scores. The article also presents improvements from its initial version based on previous reviewer feedback.

**Assessment & Comments**

**1.** **Scientific Soundness:** *Yes* – The article is scientifically sound with clear objectives, a structured methodology, and relevant findings. The use of MARS is justified and properly implemented.

**2.** **Clarity and Structure:** *Yes* – The manuscript is clearly written, well-structured, and uses standard academic language.

**Detailed Comments and Feedback**

**A.** **Strengths**

- The use of the **validated MARS tool** offers a reliable framework for assessing app quality.
- The **classification of apps** by behavioural focus is valuable and clearly operationalized.
- **Statistical correlation** between user ratings/downloads and MARS scores adds analytical strength.
- Tables are well-organized and present results in an accessible way.

**B.** **Addressed Concerns**

- The authors responded effectively to reviewer concerns from Version 1, such as:
  - Adding **source credibility** for apps in Table 2.
  - Clarifying MARS training and inter-rater reliability.
  - Including tables for correlation and chi-square analyses (now Table 5 and Table 6).
  - Rephrasing vague claims and avoiding repetition.

**C.** **Minor Remaining Points (Optional but recommended for polish)**

1. **Sample Size Limitation**
  While 20 apps were evaluated in-depth, this is a small proportion of the initial 670. The authors have acknowledged this, but a brief note on how future work could scale or diversify the dataset (e.g., inclusion of lower-download but innovative apps) would be beneficial.
2. **Clarify “Older” Apps Definition**
  The classification of app age and focus could be more explicitly defined in the **Methods** section, rather than inferred from Table 6.
3. **Discussion on Behaviour Change Techniques**
  The discussion now touches on CBT and ACT, but a slightly deeper explanation of *why* these approaches are more effective (e.g., reinforcement, coping skills, identity shift) would further strengthen the section.
4. **Limitations Section Expansion**
  The authors rightly list the limitations, but could also acknowledge that **ratings and downloads can be gamified or manipulated**, which may skew the inclusion threshold.

**Conclusion**

 This revised version of the article has adequately addressed the major concerns raised by earlier reviewers. It presents a methodologically sound, clearly written, and timely assessment of mobile apps for tobacco cessation. The authors demonstrate a thorough understanding of the MARS framework and provide practical insights for developers and public health practitioners alike.

**Final Recommendation** **:**

**Approved – No further major revisions required.**

 Optional refinements listed above may enhance impact but are not mandatory for scientific soundness.

Is the work clearly and accurately presented and does it cite the current literature?

Yes

If applicable, is the statistical analysis and its interpretation appropriate?

I cannot comment. A qualified statistician is required.

Are all the source data underlying the results available to ensure full reproducibility?

Yes

Is the study design appropriate and is the work technically sound?

Yes

Are the conclusions drawn adequately supported by the results?

Yes

Are sufficient details of methods and analysis provided to allow replication by others?

Yes

Reviewer Expertise:

Digital Health, Clinical Trial, Behaviour Change

I confirm that I have read this submission and believe that I have an appropriate level of expertise to confirm that it is of an acceptable scientific standard.

# Reviewer report: Reviewer response for version 2

**DOI:** 10.5256/f1000research.174893.r342742

**Published:** 2024-12-31

**Authors:** Cristina Rey-Reñones

**Affiliations:** 1 Research Support Unit-Camp de Tarragona, Catalan Institute of Health, Tarragona, Spain

APPROVED.

Is the work clearly and accurately presented and does it cite the current literature?

Partly

If applicable, is the statistical analysis and its interpretation appropriate?

Yes

Are all the source data underlying the results available to ensure full reproducibility?

Yes

Is the study design appropriate and is the work technically sound?

Yes

Are the conclusions drawn adequately supported by the results?

Yes

Are sufficient details of methods and analysis provided to allow replication by others?

Yes

Reviewer Expertise:

mhealth, tobacco, nursing

I confirm that I have read this submission and believe that I have an appropriate level of expertise to confirm that it is of an acceptable scientific standard.

# Reviewer report: Reviewer response for version 1

**DOI:** 10.5256/f1000research.155532.r327336

**Published:** 2024-10-18

**Authors:** Ranjani Harish, Nitika Sharma, Catherine Vincy

**Affiliations:** 1 Madras Diabetes Research Foundation, Chennai, Tamil Nadu, India

**General Comments:  **The aim of the study was to identify tobacco cessation apps on Apple and Android app stores and to categorize and analyse their features as regards to engagement, functionality, aesthetics, and information quality. Thereby recommending directions for the improvement of these apps.

**Specific Comments:**

**Title:**

1. Please specify the type of review in the title to facilitate better understanding of the article. 

**Abstract:**

1. MARS (Mobile Application Rating Scale) could have been incorporated into the keywords to enhance the specificity.

**Introduction**

1. Kindly give reference for the following lines in the introduction section: “The majority of people making independent attempts fail to maintain their quit status for even one month.”
2. The second para of this section provides statistics on tobacco cessation from India. However, the following paragraph discusses global statistics on smartphone users, which appears to be out of sync with the flow of information. To maintain coherence, it would be better to align the information properly.
3. The rationale of the review was well articulated in the introduction although a little more background on MARS and its scoring system and how it helps in heuristically evaluating the mhealth applications could be provided to strengthen the foundation of the study.

**Methodology:**

1. The introduction highlights that rankings in app stores are highly volatile and primarily influenced by app usability, meaning that an app's behaviour change potential cannot be fully assessed based on its ratings or popularity alone. However, the inclusion criteria in the methods section focus on selecting apps with a rating of 4 stars or higher and more than 10,000 downloads. Justify.
2. The inclusion of only apps with a rating of 4 stars or higher and over 10,000 downloads might exclude newer or less popular apps that could still be effective. This introduces a bias towards more established apps. Kindly justify.
3. The reviewers were trained using a 37-minute YouTube video. Please answer the following questions:
  a) It may not be sufficient for thorough, expert-level assessment using the MARS tool. Kindly comment.
  b) While a high interrater reliability (0.804) is reported, no additional details are provided on how discrepancies between reviewers were resolved. This could affect the validity of the scoring and analysis if disagreements were not consistently addressed. Justify.
  c) How reliable and authentic was the video? It is suggested to include the link for the video in the references. 
4. There’s no mention of how frequently the apps were updated or if the study accounted for updates during the research period in the methods section. App features and functionalities can change significantly with updates, which could affect the evaluation. It is suggested to include details regarding the same.
5. Less information is provided on the availability of the mhealth application, especially if is it limited to India (developed) or Indian context. This may be an important factor as some of the apps belonging to the western population could not be practical for lifestyle or behaviour change therapies among Asian Indians.
6. Statistical analyses used in the review seem basic thus undermining the actual benefits of the application and how it could be practically applied. Although the review outlines the combined effects of all the 20 applications, additional specifications or features of at least the top 5 applications could be briefed out to demonstrate how pragmatic these apps are for everyday practise.

**Results:**

1. It is recommended to provide a separate breakdown in Figure 2 for each reviewer, rather than presenting an overall summary. For example, indicate how many apps were identified by Reviewer 1 and how many were identified by Reviewer 2 using the search terms.
2. While the study title emphasizes behaviour change, "Mobile applications (apps) for tobacco cessation: Behaviour change potential and heuristic analysis using the Mobile Application Rating Scale (MARS)," the majority of the apps reviewed (14) primarily focused on goal-setting, with only 4 targeting behaviour change directly. Justify.
3. The final number of apps subjected to analysis (20 out of 670) is very small (only 3 apps from iOS). The sample size may be too small to yield reliable or meaningful statistical significance. This limits the generalizability of the findings
4. "Chi-square test was performed to analyse any association between app focus and app release dates. Older apps focused more on mere goal setting or substance use, as compared to behaviour change. Apps that have been recently developed are now focusing more on behaviour change and these results were statistically significant (P value = 0.000)."
  What is the definition of older apps? According to table 1, 50% apps were release between years 2019-2023 and an equal number were updated in 2023. This contradicts the results where the authors have mentioned that there were only 4 apps targeting behaviour change.
5. Table showing correlation and chi square test is missing.
6. It is suggested to remove pie chart as this is not adding any value to the manuscript.
7. Further explanation of statistical tests, confidence intervals, and potential confounding factors would enhance the clarity and rigor of the analysis. Additionally, providing more detail on the interpretation of correlations and the significance of app focus trends would strengthen the discussion of results.

**Discussion:**

1. This is the line from discussion section: “According to the findings of our study, the apps that focus on behaviour modification had higher user ratings and overall MARS scores with many features.”
  a) Since, authors are talking a lot about these apps. In the results section as well It is suggested to include the app names based on this categorisation in the results section for clearer reference.
  b) Also, while the study notes that most apps focussed on goal setting. There is little exploration of the implications of having a majority of apps focused on goal setting rather than behaviour change.
2. Some points mentioned in other sections of the study are repeated, such as the focus on the MARS scale and its dimensions. Kindly avoid such repetitions.
3. "Our study found more than 622 smoking cessation apps in both platforms. It is concerning that the app stores for smoking cessation applications is saturated with apps of low quality, perhaps making it difficult for users who aren't the most selective to find higher-quality apps."
  For the above statement, What criteria were used to determine low quality of an app?
4. The discussion mentions similarities with previous studies but does not provide a detailed comparison or analysis of how the current findings add to or differ from existing literature.
5. The recommendation for developing more behavior change-focused apps lacks specificity and concrete suggestions on features.
6. Kindly review the calculation for the apps excluded. The number mentioned in results (558) and the number mentioned in fig 2 (54) gives a difference of 10 which is missed out in the review. Recheck and update the table or the count.
7. While the discussion does a great job of highlighting the importance of behavior change apps, it could benefit from a deeper exploration of why behavior change techniques (e.g., cognitive-behavioral therapy, acceptance and commitment therapy) are more effective in driving smoking cessation. A more detailed explanation of the mechanisms behind these techniques, backed by literature, would strengthen the argument and provide additional value to readers who may be unfamiliar with these approaches.

**Final Comments:**

 The study aimed to evaluate tobacco cessation apps on Apple and Android app stores, analyzing their features related to engagement, functionality, aesthetics, and information quality. However, it faces issues with justifying its inclusion criteria, addressing the small sample size, and providing specific recommendations for improving behaviour change-focused apps.

Is the work clearly and accurately presented and does it cite the current literature?

Yes

If applicable, is the statistical analysis and its interpretation appropriate?

Partly

Are all the source data underlying the results available to ensure full reproducibility?

Yes

Is the study design appropriate and is the work technically sound?

Partly

Are the conclusions drawn adequately supported by the results?

Yes

Are sufficient details of methods and analysis provided to allow replication by others?

Yes

Reviewer Expertise:

NA

We confirm that we have read this submission and believe that we have an appropriate level of expertise to confirm that it is of an acceptable scientific standard, however we have significant reservations, as outlined above.

# Response

**Published:** 2024-11-15

**Authors:** Prajna Nayak

**Affiliations:** Manipal Academy of Higher Education, India

General Comments: The aim of the study was to identify tobacco cessation apps on Apple and Android app stores and to categorize and analyze their features as regards to engagement, functionality, aesthetics, and information quality. Thereby recommending directions for the improvement of these apps.

 Specific Comments:

 Title: Please specify the type of review in the title to facilitate better understanding of the article.

 Author Response : We thank the reviewer for their suggestion, the ‘abstract’ has been edited to make it clear. We could not add the type of review in the title, as it can lengthen the title further (19 words).

 Abstract:

 MARS (Mobile Application Rating Scale) could have been incorporated into the keywords to enhance the specificity.

 Author Response : We have now added it ma’am.

 Introduction

1. Kindly give reference for the following lines in the introduction section: “The majority of people making independent attempts fail to maintain their quit status for even one month.”
2. The second para of this section provides statistics on tobacco cessation from India. However, the following paragraph discusses global statistics on smartphone users, which appears to be out of sync with the flow of information. To maintain coherence, it would be better to align the information properly.
3. The rationale of the review was well articulated in the introduction although a little more background on MARS and its scoring system and how it helps in heuristically evaluating the mhealth applications could be provided to strengthen the foundation of the study.

Author Response :

1. We have now added the reference, ma’am.
2. .We have now provided India statistics to synchronize with the tobacco cessation statistics.
3. We have now provided details on the MARS scale. More details are available in the methodology section ma’am.

Methodology:

1. The introduction highlights that rankings in app stores are highly volatile and primarily influenced by app usability, meaning that an app's behavior change potential cannot be fully assessed based on its ratings or popularity alone. However, the inclusion criteria in the methods section focus on selecting apps with a rating of 4 stars or higher and more than 10,000 downloads. Justify.
2. The inclusion of only apps with a rating of 4 stars or higher and over 10,000 downloads might exclude newer or less popular apps that could still be effective. This introduces a bias towards more established apps. Kindly justify.
3. The reviewers were trained using a 37-minute YouTube video. Please answer the following questions:
  a) It may not be sufficient for thorough, expert-level assessment using the MARS tool. Kindly comment.
  b) While a high interrater reliability (0.804) is reported, no additional details are provided on how discrepancies between reviewers were resolved. This could affect the validity of the scoring and analysis if disagreements were not consistently addressed. Justify.
  c) How reliable and authentic was the video? It is suggested to include the link for the video in the references. 
4. There’s no mention of how frequently the apps were updated or if the study accounted for updates during the research period in the methods section. App features and functionalities can change significantly with updates, which could affect the evaluation. It is suggested to include details regarding the same.
5. Less information is provided on the availability of the mhealth application, especially if is it limited to India (developed) or Indian context. This may be an important factor as some of the apps belonging to the western population could not be practical for lifestyle or behavior change therapies among Asian Indians.
6. Statistical analyses used in the review seem basic thus undermining the actual benefits of the application and how it could be practically applied. Although the review outlines the combined effects of all the 20 applications, additional specifications or features of at least the top 5 applications could be briefed out to demonstrate how pragmatic these apps are for everyday practice.

Author Response : 

1. We have now added the justification in the 1 ^st^ para of Discussion, ma’am.
2. We have now added the justification in the 1 ^st^ para of Discussion and also mentioned in the limitations, ma’am.
3. Since the video was uploaded by the developer of the scale himself, we used it as a reference for understanding the scale better. We have also added on how the discrepancies were resolved in the Methods section. (13) MARS training video - YouTube , we have now added the link to the video, ma’am.
4. As the study was conducted in a relatively short period of time, we have not mentioned updates during study period.
5. Since the Apple and Android App stores both show all the apps, irrespective of the country that developed the app, we could not isolate the Indian apps. Also, we did not have access to numerous international apps created exclusively for use within each nation as phone numbers were required for registration and app usage.
6. As this could lengthen the manuscript, we could not brief on top 5 apps. Instead, we have tried to summarize the results of top 20 apps distinctly.

Results:

1. It is recommended to provide a separate breakdown in Figure 2 for each reviewer, rather than presenting an overall summary. For example, indicate how many apps were identified by Reviewer 1 and how many were identified by Reviewer 2 using the search terms.
2. While the study title emphasizes behavior change, "Mobile applications (apps) for tobacco cessation: Behavior change potential and heuristic analysis using the Mobile Application Rating Scale (MARS)," the majority of the apps reviewed (14) primarily focused on goal-setting, with only 4 targeting behavior change directly. Justify.
3. The final number of apps subjected to analysis (20 out of 670) is very small (only 3 apps from iOS). The sample size may be too small to yield reliable or meaningful statistical significance. This limits the generalizability of the findings
4. "Chi-square test was performed to analyze any association between app focus and app release dates. Older apps focused more on mere goal setting or substance use, as compared to behavior change. Apps that have been recently developed are now focusing more on behavior change and these results were statistically significant (P value = 0.000)."
  What is the definition of older apps? According to table 1, 50% apps were release between years 2019-2023 and an equal number were updated in 2023. This contradicts the results where the authors have mentioned that there were only 4 apps targeting behavior change.
5. Table showing correlation and chi square test is missing.
6. It is suggested to remove pie chart as this is not adding any value to the manuscript.
7. Further explanation of statistical tests, confidence intervals, and potential confounding factors would enhance the clarity and rigor of the analysis. Additionally, providing more detail on the interpretation of correlations and the significance of app focus trends would strengthen the discussion of results.

Author Response : 

1. Though both authors downloaded, selected and excluded apps together and then reviewed each app independently.
2. Since we had to unbiasedly select the apps out of 54 apps, we included only the highly rated and most downloaded apps. Focus of the apps were checked later during the study.

Nevertheless, other 14 apps, though had primary focus as goal setting, also had behavior counselling in their content. Hence, we have included them.

1. Though we initially got 670 apps, the actual tobacco cessation apps were only 54. (Figure 2 flowchart for app identification). Hence, 20 out of 54 were selected, ma’am.
2. We have now added a table mentioning the app release dates and app primary focus. (Table 6).
3. We have now added it ma’am (Table 5)
4. We have removed the pie chart now.
5. We have now mentioned.

Discussion:

1. This is the line from discussion section: “According to the findings of our study, the apps that focus on behavior modification had higher user ratings and overall MARS scores with many features.”
  a) Since, authors are talking a lot about these apps. In the results section as well It is suggested to include the app names based on this categorization in the results section for clearer reference.
  b) Also, while the study notes that most apps focused on goal setting. There is little exploration of the implications of having a majority of apps focused on goal setting rather than behavior change.
2. Some points mentioned in other sections of the study are repeated, such as the focus on the MARS scale and its dimensions. Kindly avoid such repetitions.
3. "Our study found more than 622 smoking cessation apps in both platforms. It is concerning that the app stores for smoking cessation applications is saturated with apps of low quality, perhaps making it difficult for users who aren't the most selective to find higher-quality apps."
  For the above statement, What criteria were used to determine low quality of an app?
4. The discussion mentions similarities with previous studies but does not provide a detailed comparison or analysis of how the current findings add to or differ from existing literature.
5. The recommendation for developing more behavior change-focused apps lacks specificity and concrete suggestions on features.
6. Kindly review the calculation for the apps excluded. The number mentioned in results (558) and the number mentioned in fig 2 (54) gives a difference of 10 which is missed out in the review. Recheck and update the table or the count.
7. While the discussion does a great job of highlighting the importance of behavior change apps, it could benefit from a deeper exploration of why behavior change techniques (e.g., cognitive-behavioral therapy, acceptance and commitment therapy) are more effective in driving smoking cessation. A more detailed explanation of the mechanisms behind these techniques, backed by literature, would strengthen the argument and provide additional value to readers who may be unfamiliar with these approaches.

Author Response : 

1. We have now named those apps focusing on behavior change in the results section. Although the primary focus of majority of apps was goal setting, they also had behavior modification components in them.
2. We have now removed the repetitions, ma’am.
3. Most of the other apps had mere goal setting or basic details of health affects before and after quitting tobacco. Hence we had mentioned like so. We thank the reviewers for drawing attention on this, and we have now corrected the sentence.
4. We have now added.
5. We have now added ma’am.
6. We have corrected it.
7. We have now briefly explained important behavior change techniques for smoking cessation.

# Reviewer report: Reviewer response for version 1

**DOI:** 10.5256/f1000research.155532.r236779

**Published:** 2024-01-25

**Authors:** Cristina Rey-Reñones

**Affiliations:** 1 Research Support Unit-Camp de Tarragona, Catalan Institute of Health, Tarragona, Spain

Dear Author's,

 I trust this message finds you well. I recently read your article titled "Mobile Applications (Apps) for Tobacco Cessation: Behavior Change Potential and Heuristic Analysis Using the Mobile Application Rating Scale (MARS) [Version 1; Peer Review: Awaiting Peer Review]." I commend you on addressing a pertinent contemporary need in your research.

 The work is presented with clarity and precision. Notably, a total of 21 bibliographic citations are included, with 13 (61%) sourced from the last five years.

 In light of the commendable effort, I would like to offer a couple of constructive suggestions for consideration. Firstly, it would be beneficial to specify which of the applications studied have scientific evidence supporting their efficacy, effectiveness, and efficiency. This additional detail would enhance the comprehensiveness of your analysis.

 Additionally, I would like to suggest broadening the scope of your study by incorporating other forms of tobacco consumption, such as "vaping" or "e-cigarette," in addition to traditional tobacco use. This expansion would contribute to a more comprehensive understanding of the subject matter.

 Once again, congratulations on your valuable contribution to the field. I appreciate your dedication to advancing research in this area.

 Kind regards, 

Is the work clearly and accurately presented and does it cite the current literature?

Partly

If applicable, is the statistical analysis and its interpretation appropriate?

Yes

Are all the source data underlying the results available to ensure full reproducibility?

Yes

Is the study design appropriate and is the work technically sound?

Yes

Are the conclusions drawn adequately supported by the results?

Yes

Are sufficient details of methods and analysis provided to allow replication by others?

Yes

Reviewer Expertise:

mhealth, tobacco, nursing

I confirm that I have read this submission and believe that I have an appropriate level of expertise to confirm that it is of an acceptable scientific standard, however I have significant reservations, as outlined above.

# Response

**Published:** 2024-11-15

**Authors:** Prajna Nayak

**Affiliations:** Manipal Academy of Higher Education, India

We thank the reviewer for their suggestions, we have now made the following corrections:

 1. As mentioned by the reviewer to add scientific evidence available for the applications studied, we have now added a column in table 2 on the sources of information if available. This will enable readers on the legitimacy of the source.

 2. We could not add the incorporate other forms of tobacco consumption, such as "vaping" or "e-cigarette," in addition to traditional tobacco use. Because, we had not used these terms in our ‘Search strategy’. Addition of these would have resulted in apps designed to assist in quitting e-cigarettes too. Hence, we are afraid we will not be able to incorporate them, now.
